# Genetic causes of isolated short stature

**DOI:** 10.20945/2359-3997000000105

**Published:** 2019-02-01

**Authors:** Gabriela A. Vasques, Nathalia L. M. Andrade, Alexander A. L. Jorge

**Affiliations:** 1 Universidade de São Paulo Universidade de São Paulo Hospital das Clínicas Faculdade de Medicina São Paulo SP Brasil Unidade de Endocrinologia Genética (LIM25), Hospital das Clínicas, Faculdade de Medicina, Universidade de São Paulo (HCFMUSP), São Paulo, SP, Brasil; 2 Universidade de São Paulo Universidade de São Paulo Hospital das Clínicas Laboratório de Hormônios e Genética Molecular São Paulo SP Brasil Unidade de Endocrinologia do Desenvolvimento, Laboratório de Hormônios e Genética Molecular (LIM42), Hospital das Clínicas, Faculdade de Medicina, Universidade de São Paulo (HCFMUSP), São Paulo, SP, Brasil

**Keywords:** Short stature, growth, growth cartilage, growth hormone

## Abstract

Short stature is a common feature, and frequently remains without a specific diagnosis after conventional clinical and laboratorial evaluation. Longitudinal growth is mainly determined by genetic factors, and hundreds of common variants have been associated to height variability among healthy individuals. Although isolated short stature may be caused by the combination of variants, with a deleterious impact on the growth of individuals with polygenic inheritance, recent studies have pointed out some monogenic defects as the cause of the growth disorder observed in nonsyndromic children. The majority of these defects are in genes related to the growth plate cartilage and in the growth hormone (GH) – insulin-like growth factor 1 (IGF-1) axis. Affected patients usually present the mildest spectrum of some forms of skeletal dysplasia, or subtle abnormalities of laboratory tests, suggesting hormonal resistance or insensibility. The lack of specific characteristics, however, does not allow formulation of a definitive diagnosis without the use of broad genetic studies. Thus, molecular genetic studies including panels of genes or exome analysis will become essential in investigating and identifying the causes of isolated short stature in children, with a crucial impact on treatment and follow-up.

## INTRODUCTION

Short stature is defined as a condition characterized by height more than 2 standard deviation scores below the mean observed in age and sex control population (height SDS < −2) (1). Based on this definition, short stature affects 2.3% of children, and is a matter of concern for many parents seeking medical attention. Although growth disorder may be a clinical presentation of an underlying disease, in around 70% of the cases, short stature is not associated with any clinical and laboratory evidence that justify growth impairment (2). These children are usually classified as having constitutional or idiopathic short stature (ISS) (3). In this review, we also used the term isolated short stature, in view of the growing number of monogenic conditions that explain the short stature phenotype in nonsyndromic children.

Height is one of the human traits with a higher degree of heritability (> 80%), which means that genetic variability is the main determinant of stature (4). Up to date, more than 600 common variants (with allele frequencies greater than 1%) distributed across more than 400 regions of the genome have been independently associated with height (5). The impact of each identified common variant on stature is small (around 1-4 mm). More recently, studies have pointed out that rare variants (frequency < 1%) may exert an effect on height variability 10 to 20 times greater than that exert by common variants (about 20 mm per allele). Many of these common and rare variants are in genes associated with syndromic growth disorders or in genes involved in the development of the growth cartilage (6).

Since short stature is a common clinical presentation, it is widely accepted that it is caused by common variants with polygenic inheritance (7,8). However, recent studies have challenged this dogma, and proposed that many of the children classified as ISS could instead have a monogenic defect. A part of this group would represent the mildest spectrum of phenotypic variability of syndromic conditions (9,10). For example, there are short stature individuals harboring pathogenic variants in genes responsible for the Noonan syndrome who do not present any of the other phenotypic alterations that would allow the recognition of this condition clinically (11). Additionally, genes that regulate the growth plate and that had already been associated with skeletal dysplasias were recently pointed as responsible for some cases of ISS (12–16). This review provides an update on monogenic causes of isolated short stature.

## GENES THAT REGULATE GROWTH PLATE

Endochondral ossification is a complex process which occurs in the growth plate to promote bone elongation and consequent increase in height. It involves proliferation, hypertrophy and senescence of chondrocytes and also cartilage matrix synthesis (17). Paracrine and autocrine factors are the main regulators of endochondral ossification and defects in genes that encode or disrupt these factors (summarized in Table 1) have been associated with ISS. Each of these genes is responsible for a small proportion of cases (up to 2%), but this proportion may be significantly higher in familial short stature. Defects in genes that regulate growth plate, inherited in an autosomal dominant manner, cause a variable phenotype in terms of degree of short stature and body proportions. The atypical radiological findings do not allow the precise diagnosis and some of the patients are recognized as having a subclinical skeletal dysplasia.

**Table 1:** Genes involved in the endochondral ossification process that are associated with isolated short stature

Gene	First report in patient classified as ISS	Frequency in ISS (%)	Additional common findings	References
*SHOX*	1997	2.6 (1.1 to 22.2)	Mild abnormal body proportion, family member with Madelung deformity	(18)
*NPR2*	2013	1.8 to 6 (13.6*)	Nonspecific skeletal abnormalities, as short metacarpals	(13,22)
*NPPC*	2018	NA	Two families described with brachydactyly	(16)
*ACAN*	2014	1.4	Accelerated bone maturation and early-onset osteoarthritis	(52,53)
*IHH*	2018	3.4	Shortening of the middle phalanx of the 5th finger	(15,54)
*FGFR3*	2015	NA	Only one family described with normal body proportion	(32)

* Familial cases; NA: not available; ISS: idiopathic short stature.

### SHOX

**Figure 1:**
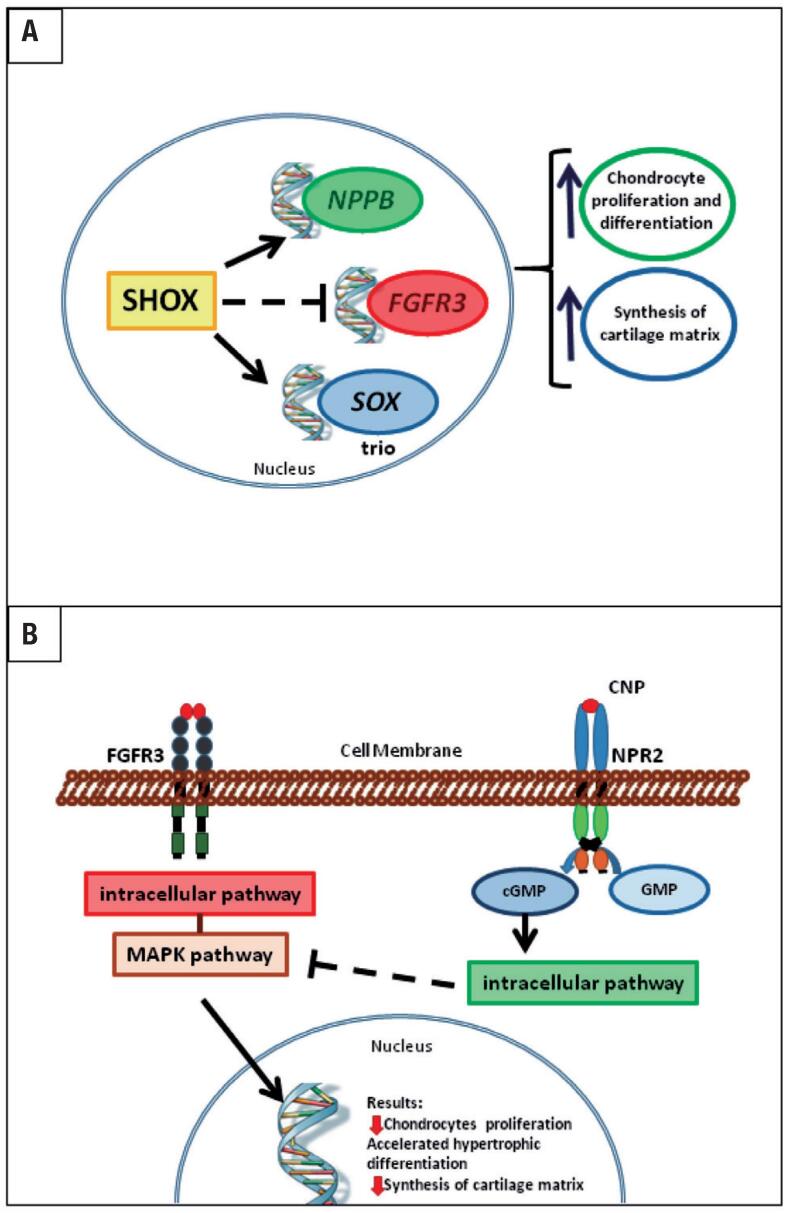
Schematic representation of chondrogenesis regulation by genes associated to isolated short stature phenotype. (A) SHOX is a transcriptional factor that enhances *NPPB* and *SOX* trio expression and inhibits *FGFR3* expression. This leads to an increase in chondrocyte proliferation and differentiation and an increase in cartilage matrix synthesis. (B) CNP and FGF signaling pathways converge at the MAPK pathway. The opposite effect of CNP on FGFR3 pathway improves longitudinal bone growth by increasing chondrocyte proliferation and differentiation and cartilage matrix synthesis.

The short stature homeobox (*SHOX*) gene is located in the pseudoautosomal region 1 of both sex chromosomes and is expressed in growth cartilage, especially in hypertrophic chondrocytes. The role of the *SHOX* gene as a regulator of the growth plate has not been fully understood. It is known that SHOX is a transcription factor that increases *NPPB* and inhibits *FGFR3* expression, respectively (Figure 1A). Both effects stimulate and coordinate chondrocytes proliferation and differentiation in order to promote longitudinal growth. In addition, SHOX interacts with the SOX trio (*SOX9, SOX5* and *SOX6* genes), which has an important role in cartilage matrix synthesis (18). The causative relation between *SHOX* defects and ISS was first described in 1997 (12). Since then, *SHOX* haploinsufficiency has become the main recognized monogenic cause of short stature, being responsible for 2.6% of the nonsyndromic cases of short stature. Homozygous defects in *SHOX* gene cause Langer mesomelic dysplasia, a rare skeletal dysplasia with severe short stature and limb aplasia or hypoplasia of the ulna and fibula. Heterozygous *SHOX* haploinsufficiency causes phenotypes that range from isolated short stature to the complete picture of the Leri-Weill dyschondrosteosis (LWD), a skeletal dysplasia characterized by mesomelia and Madelung deformity (*i.e.* shortening and bowing of the radius with dorsal subluxation of the distal ulna) (19). Around 80% of reported *SHOX* defects are deletions involving the gene or regulatory regions, with the remaining ones being point mutations. Although no genotype-phenotype correlation has been identified in individuals with *SHOX* deletions encompassing exons and point mutations, deletion in downstream *SHOX* enhancer have been associated with a milder phenotype (18).

Abnormal body proportion, defined by the [sitting height]/[height] ratio for age and sex (SH/H SDS) > 2, is present in the vast majority of children with isolated short stature caused by *SHOX* haploinsufficiency (20). The degree of short stature is variable, and the mean growth deficit of affected individuals is of around 12 cm, which means that there are carriers with height within the normal range.

A reasonable clinical selection criterion to screen for *SHOX* defects in a child with isolated short stature is the presence of abnormal body proportion, especially if the phenotype of disproportional short stature segregates in an autosomal dominant manner and/or there is a family member with Madelung deformity. Particular attention should be given to the regulatory regions of *SHOX* gene, since deletions located outside the coding region are more commonly associated with short stature without other specific findings.

### NPR2 and NPPC

**Figure 2:**
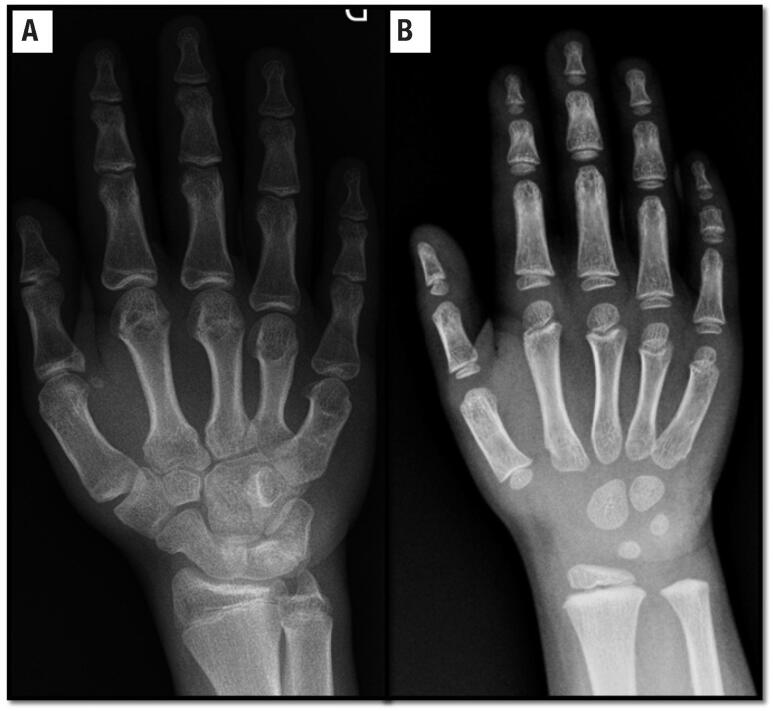
Hand radiographs of children carrying *NPR2* and *IHH* mutations. (A) Hand radiograph of a patient with heterozygous mutation in *NPR2.* Shortening of metacarpal is clearly observed. (B) Hand radiograph of a child with heterozygous mutation in *IHH* showing the shortening of the middle phalanx of the 5^th^ finger with cone-shaped epiphyses.

The C-type natriuretic peptide (CNP) and its receptor (NPR-B) are important regulators of the endochondral ossification process (21). CNP and NPR-B are encoded by *NPPC* and *NPR2* genes, respectively. They are notably expressed in the hypertrophic zone of the growth plate. The CNP/NPR-B system stimulates chondrocyte proliferation and differentiation and synthesis of cartilage matrix in an autocrine/paracrine manner. The molecular mechanism that explains these actions is at least partially identified and involves the inhibition of the FGFR3 pathway (Figure 1B). After CNP binding to NPR-B, there is an intracellular accumulation of cyclic GMP (cGMP), that activates cGMP-dependent protein kinases I and II (cGKI and cGKII). cGKII inhibits the activation of RAF-1. Biallelic loss-of-function mutations in *NPR2* cause acromesomelic dysplasia type Maroteaux (21). Heterozygous *NPR2* mutations in a cohort of ISS children were first described in 2013 (13). Since then, other studies have replicated this finding and the prevalence of heterozygous *NPR2* mutations in ISS patients has ranged from 1.8% to 13.6% (in familial cases) (22). Similar to what has been observed in patients with *SHOX* haploinsufficiency, carriers of *NPR2* mutation have a variable degree of short stature and some carrier relatives presented height at the lower limit of the normal range. Abnormal body proportion (SH/H SDS > 2) and nonspecific skeletal abnormalities, as short metacarpals (Figure 2A), were also observed among patients with *NPR2* mutations. Due to the phenotypic heterogeneity, there is, at the moment, no clinical feature that enables establishing a criterion to select short stature patients for for *NPR2* molecular-genetic screening.

More recently, heterozygous *NPPC* mutations have been identified in two families with ISS. In total, six *NPPC* carriers were identified, two being Brazilian and four Spanish. Their height SDS ranged from −4.3 to −2.3 and all of them also had small hands phenotype (16).

### ACAN

The Aggrecan gene (*ACAN*) encodes a proteoglycan present in the extracellular matrix. Aggrecan has a fundamental structural and functional role in the growth plate cartilage. Initially, *ACAN* mutations were associated with two rare types of skeletal dysplasias, one with an autosomal dominant (Spondyloepiphyseal dysplasia, Kimberley type) and another with a recessive inheritance (Spondyloepimetaphyseal dysplasia, aggrecan type) (23). In 2014, heterozygous *ACAN* mutations were identified as cause of short stature in three families with no skeletal findings suggestive of skeletal dysplasia. Children presented accelerated bone maturation, even before puberty, and some subjects had accompanying early-onset osteoarthritis (14). This dual phenotypic presentation occurs because aggrecan is a component of both growth plate and articular cartilage.

Since the first report, more than one hundred individuals from more than twenty families with autosomal dominant inherited short stature were identified as carriers of *ACAN* mutations (24). Affected children seem to have growth impairment even before birth, and mutations in this gene were reported in a cohort of short children born small for their gestational age, mainly regarding birth length (25). The majority of children with *ACAN* mutations have proportionate short stature with advanced bone age. Due to the poor pubertal spurt followed by early growth cessation, final height is still more compromised and adults present lower height SDS and, frequently, body disproportion. Other clinical features recognized in affected subjects are brachydactyly, mild midface hypoplasia, and flat nasal bridge. Some individuals present early-onset osteoarthritis, which appears until fourth decade of life and affects most commonly the knees, with a variable degree of severity (24). Whereas most children from cohorts of patients with ISS have delayed bone age, *ACAN* mutation should be suspected in a short child with advanced bone age.

### IHH

The Indian hedgehog gene (*IHH*) is expressed in prehypertrophic chondrocytes of the growth plate, and codifies a key paracrine regulator of endochondral ossification, which coordinates chondrocytes proliferation and differentiation (26). Similar to what occurs with the other genes that regulate growth plate described above, *IHH* defects had already been recognized as a cause of two skeletal dysplasias. Homozygous *IHH* mutations cause acrocapitofemoral dysplasia, characterized by severe disproportionate short stature with cone-shaped epiphyses in hands and hips. Additionally, heterozygous mutations in *IHH* cause brachydactyly type A1 (BDA1), characterized by a striking shortening of the middle phalanges, which can be fused with the terminal ones (27). Interestingly, short stature was not consistently reported in patients with BDA1 (28).

In 2018, we described heterozygous *IHH* mutations as a cause of the growth impairment in an ISS cohort (frequency of 3.4%) (15). Our patients with heterozygous *IHH* variants did not present classical features of BDA1. The only recurrent radiological finding observed was a varying degree of shortening of the middle phalanx of the 5^th^ finger (Figure 2B), which is the defining feature of Brachymesophalangia – V (BMP-V). BMP-V was observed in 64.3% hand radiographs from individuals with heterozygous mutations in *IHH* in our cohort, which was significantly higher than that observed in a population study (12.1%) (15). Four probands were born small for gestational age considering only length at birth. Affected subjects typically had mild disproportional short stature. The severity of short stature is variable and disproportionality seems to become more pronounced over the years.

### FGFR3

The fibroblast growth factor receptor-3 (FGFR3) pathway acts as a negative regulator of the growth plate chondrogenesis (Figure 1). Heterozygous gain-of-function mutations in the *FGFR3* cause achondroplasia and hypochondroplasia. Achondroplasia is the most frequent skeletal dysplasia and is clinically characterized by severe disproportional short stature with rhizomelia (29). In contrast, hypochondroplasia is generally less severe and presents a broader phenotypic variability (30).

Although there is an evident abnormal proportion in patients with hypochondroplasia and *FGFR3* alterations is rare in patients classified as ISS (31), some children still can be undiagnosed at first evaluation. More recently, a study identified *FGFR3*-activating mutation causing autosomal dominant familial proportionate short stature without other specific findings (32).

## GENES RELATED TO THE GH-IGF1 AXIS

Since the growth hormone (GH) and insulin-like growth factor 1 (IGF-1) have been initially recognized as the main regulators of longitudinal growth, the first researches in the field of genetics of short stature attempted to identify children with defects in the GH–IGF-1–bone axis. GH is secreted by the pituitary gland, and promotes bone elongation mainly through regulating synthesis of IGF-1, both circulating and peripheral. Growth impairment can be caused by gene defects that affect several components of this cascade (summarized in Table 2), from the synthesis of GH to the action of IGF-1. Affected subjects may not present classical features of GH/IGF-1 deficiency or resistance, in which cases they would often be classified as having ISS.

**Table 2:** Genes related to GH-IGF1 axis that are associated with isolated short stature

Gene	First report in patient classified as ISS	Frequency in ISS (%)	Inheritance	Additional common findings	References
*GH1*	2003	NA	AD	Low height velocity and delayed bone age	(35)
*GHSR*	2006	2.0 to 2.4	AD/AR	GHD and ISS in the same family	(37,55)
*GHR*	1995	0 to 5.0	AD	Laboratory suggestive of partial GH insensitivity	(56,57)
*STAT5B*	2018	NA	AD	Three families described with eczema and laboratory suggestive of partial GH insensitivity	(41)
*IGF1*	2012	NA	AD	Birth weight and birth length in the lower normal range	(45,58)
*IGF1R*	2003	1.6 to 2.0	AD	Born small for gestational age in the most cases and elevated levels of IGF-1	(46,47,59)
*IGFALS*	2004	NA	AR	Severe deficiency of IGF-1 and IGFBP-3 disproportional to the severity of short stature	(48)
*PAPP-A2*	2016	NA	AR	High levels of IGF-1 and IGFPB-3	(50)

NA: prevalence study is not available; ISS: idiopathic short stature.

### GH1

Defects in *GH1* gene, which encodes GH, were firstly recognized as a cause of isolated GH deficiency (IGHD). The genetic defects responsible for this phenotype are generally deletions involving the gene or point mutations leading to a truncated protein, although some missense mutations and rare promoter region mutations have already been reported (33). Affected children typically present proportionate short stature with low growth velocity, low IGF-1 concentration and an inadequate response during GH provocative tests. Additionally, heterozygous and homozygous mutations in *GH1* have been associated with bioinactive GH, characterized by a similar phenotype to IGHD, except for normal or elevated GH levels (34).

Children with *GH1* defects may present a milder phenotype than that classically described, with no typical findings that allow the diagnosis of IGHD or of bioinactive GH. This may be particularly important in less severe genetic defects, as reported in a study that identified a heterozygous mutation in the *GH1* gene promoter associated to ISS (35). In these cases, the etiology of short stature remains undefined after the initial clinical and laboratorial evaluation.

### GHSR

Two peptides stimulate GH synthesis and secretion: growth hormone-releasing hormone (GHRH) and ghrelin. Ghrelin acts through interaction with the GH secretagogue receptor (GHSR) and has also a potent orexigenic effect (36).

Defects in the *GHSR* gene have been associated with ISS and GH deficiency with variable severity and penetrance. Both phenotypes were even described in the same family (37). Additionally, some affected children had delayed puberty (38). This heterogeneity in clinical presentation may be partially explained because gene defects can result in protein alterations that affect the binding affinity to ghrelin and the constitutive activity of the receptor.

### GHR

GH exerts its action by binding to the GH receptor (GHR), a member of the cytokine superfamily of receptors. Homozygous or compound heterozygous mutations in *GHR* gene cause complete GH insensitivity (GHI), known as Laron syndrome, characterized by extreme short stature, high or normal GH concentrations, very low IGF-1 and IGFBP-3 levels, and no increase in IGF-1 concentration after exogenous GH stimulation (39). Differently, heterozygous *GHR* mutations can cause a variable spectrum of GHI, ranging from total absence of GHR activation to milder impairment causing subtler clinical phenotypes.

Until now, only seven patients have been reported with heterozygous *GHR* mutations with a dominant-negative effect causing short stature with recognizable features of partial GHI. Several other studies have reported heterozygous *GHR* variants in ISS patient cohorts, with a frequency between 5% and 15.5%. However, many of these variants have not been proven to be causative of short stature. It is supposed that the ISS phenotype, accompanied by some degree of GHI, may be caused by less deleterious heterozygous *GHR* mutations. Some authors suggested that low growth hormone binding protein (GHBP) levels could indicate a defect in the extracellular domain of the GHR in these patients (40).

Hence, since the clinical characterization remains subjective, heterozygous *GHR* mutations may be responsible for the growth failure observed in a group of children with isolated short stature and non-classical laboratory phenotype associated with impaired GH action.

### STAT5B

STAT5B is an essential protein in the intracellular signaling pathway downstream of GHR activation. Homozygous inactivating *STAT5B* mutations cause the classical phenotype of GH insensitivity associated with immunodeficiency (mainly eczema and chronic pulmonary disease). Relatives of autosomal recessive *STAT5B* deficient patients who carry *STAT5B* mutations usually have heights within the low normal range (41).

In 2018, three heterozygous *STAT5B* mutations with a dominat-negative effect were described in eleven individuals from three unrelated families, whose probands had undiagnosed short stature and laboratory evaluation of mild GH insensitivity. Most of the affected individuals presented elevated IgE, mild eczema and short stature with inter- and intra-familial variability (42).

### IGF1 and IGF1R

IGF-1 is the main growth factor in intrauterine development and in postnatal growth and acts through the binding to a cell surface tyrosine kinase receptor called IGF1R. Deletions or homozygous loss-of-function mutations in *IGF1* gene cause severe pre- and post-natal growth failure, microcephaly and retarded intellectual development (43). Affected patients present extreme low IGF-1 levels with normal or high GH concentrations. Individuals heterozygous for *IGF1* variants were significantly shorter and had reduced head circumferences compared to noncarrier relatives. Affected children born from affected mothers appear to have a more compromised height, which suggest a role of placental dysfunction (44).

In 2014, a complete *IGF1* gene deletion in heterozygosity was reported in a patient with ISS. The affected child had birth length and weight within the low normal value and presented postnatal growth failure with low-normal serum IGF-1 (45).

Patients heterozygous for *IGFR1* defects have a similar phenotype to that of patients with *IGF-1* mutations, except for the relatively high IGF-1 concentrations (46). Although this is a rare condition, some patients may have a less obvious clinical presentation, and short statute may be the only recognizable characteristic (47).

### Ternary complex defects (IGFALS and PAPP-A2)

Most of the serum IGF-1 circulates bound to IGF binding protein type 3 (IGFBP-3) and acid-labile subunit (ALS), forming a ternary complex. This complex is essential to extend serum IGF-1 half-life and to decrease its bioavailability at tissue level. On the other hand, pregnancy-associated plasma protein A2 (PAPPA2) is a serum and tissue protease responsible for the proteolysis of IGFBP-3, releasing IGF-1 from the ternary complex.

Biallelic loss-of-function mutations in *IGFALS* result in extreme decrease in the circulating levels of functional ALS (48). The consequence of this is the impossibility to form a ternary complex with reduced IGF-1 and IGFBP-3 levels, which is reflected clinically in mild short stature associated with delayed puberty. Over the past years, it has been postulated that heterozygous variants in *IGFALS* are responsible for isolated short stature phenotype. Affected children presented partial ALS deficiency and a mean decrease in height of 1 SDS (49).

The opposite phenomenon, *i.e.* increased IGF-1 and IGFBP-3 levels, was first described in 2016. Five affected children coming from two different families had PAPPA2 deficiency caused by autosomal recessive mutations in *PAPPA2* gene. In addition to variable short stature (heights ranged from −1.0 to −3.8 SDS), some patients had microcephaly, thin long bones, low bone mineral density, and insulin resistance (50).

## OTHER GENE

### PTPN11

The protein-tyrosine phosphatase non-receptor type 11 gene (*PTPN11*) encodes the non-receptor Src-homology 2 (SH2) domain-containing protein tyrosine phosphatase 2 (SHP2). SHP2 participates in multiple intracellular signaling pathways, including the Ras/MAPK cascade. Activating heterozygous mutations in *PTPN11* cause the Noonan syndrome (NS), characterized by reduced postnatal growth, congenital heart disease, and facial dysmorphisms. Commonly, affected children present low serum IGF-1 level, which reflects some degree of GH insensitivity (51).

Similarly to the other genes reported above, there is a variable clinical presentation in affected individuals, even within the same family. In addition, the craniofacial features change with age, therefore it can be sometimes challenging to make a clinical diagnosis of NS. Finally, pathogenic variants in *PTPN11* have been identified in patients from a cohort of ISS children (11).

## CONCLUSION AND FUTURE DIRECTIONS

Over the past several years, an increasing number of genetic variants have been associated with short stature. Many of these genes have been long known to be causing extreme phenotypes of growth disorders, which are clinically recognized, such as skeletal dysplasias or GH resistance syndromes. Nowadays, with the advent and greater availability of next-generation sequencing techniques, it has become possible to identify several gene defects in short stature children that presented clinically with the mildest spectrum of the diseases and that were initially classified as ISS.

The identification of the genetic causes of short stature prevents children to undergo unnecessary exams, and can give hints on the growth outcomes, with or without recombinant human GH (rhGH). For example, it is known that patients with *SHOX* defects have a good response to rhGH therapy (18). Conversely, it is expected that ISS children with less severe defects of the GH-IGF-1 axis, including less severe *GHR* mutations, will respond less to rhGH use at regular dosage (40). Besides that, a CNP analog named vosoritide, that has been used as an experimental drug for the treatment of achondroplasia, can be in the future an option to treat children with nonsyndromic short stature, especially those with heterozygous mutations in the *NPR2* or in the *NPPC* genes (21).

The lack of specific characteristics makes accurate diagnosis difficult without the use of molecular genetic study. For this same reason, candidate gene analysis is generally not sufficient, and hence, a multiple-gene testing approach using next-generation sequencing (NGS) is preferable. The choice of the use of whole exome sequencing (WES) or gene-panel analysis of the genes will depend on the availability and on cost-benefit evaluation. Even when using the WES, the analysis of these patients should prioritize genes already associated with the isolated short stature phenotype. In the years that will follow, molecular genetic study using a panel of genes or exome analysis will become mandatory when investigating children with isolated short stature, with a crucial impact on treatment and follow-up.
